# Upregulation of DNA repair-related genes in the prefrontal cortex of patients with schizophrenia with low genetic risk

**DOI:** 10.1038/s41537-026-00748-9

**Published:** 2026-04-08

**Authors:** Kazusa Miyahara, Mizuki Hino, Risa Shishido, Atsuko Nagaoka, Hideomi Hamasaki, Akiyoshi Kakita, Hiroaki Tomita, Yasuto Kunii

**Affiliations:** 1 Department of Disaster Psychiatry, International Research Institute of Disaster Science, Tohoku University, Sendai, Japan; 2 Department of Neuropsychiatry, School of Medicine, Fukushima Medical University, Fukushima, Japan; 3 Department of Psychiatry, Tohoku University Hospital, Tohoku, Japan; 4 Department of Pathology, Brain Research Institute, Niigata University, Niigata, Japan; 5 Department of Psychiatry, Graduate School of Medicine, Tohoku University, Tohoku, Japan

**Keywords:** Schizophrenia, Schizophrenia

## Abstract

Schizophrenia is a heterogeneous disorder, with subpopulations showing a relatively higher heritable predisposition based on many common genetic variants with minimal effects, whereas other subpopulations likely have alternative pathogenic backgrounds, including rare genetic variants with large effects. These heterogeneities may hinder the identification of molecular profiles related to the disorder’s pathogenesis. Therefore, this study aimed to identify transcriptional profiles specific to patients with schizophrenia with high heritable predisposition, indicated by high polygenic risk scores (PRS), and an alternative subgroup with low PRS. RNA-seq-based transcriptome data of the prefrontal cortices were compared among subgroups of patients with high PRS (PRS at or above the median; *n* = 12), low PRS (PRS below the median; *n* = 11), and controls (*n* = 21). Gene-category enrichment analysis of 584 differentially expressed genes (DEGs) identified 8 DEGs associated with DNA repair. Additionally, the expression levels of these DNA repair-related genes were associated with the general psychopathology scale, raising the hypothesis that oxidative stress accumulation, indicated by superoxide dismutase 2 expression may contribute to DNA repair activation. Furthermore, the expression levels of six DNA repair-related genes were significantly linked to the severity of the general psychopathology scale, suggesting that DNA repair might affect the clinical phenotypes of schizophrenia. This study used PRS to stratify patients with schizophrenia, highlighting the potential role of DNA repair-related pathways to the heterogeneity of schizophrenia. Understanding the role of DNA repair could lead to personalized treatments that target oxidative stress-related molecules.

## Introduction

Schizophrenia is a highly heritable and heterogeneous disorder^1,2^, with variations spanning clinical phenotypes—such as dimension and severity of symptoms^3^ and pharmaco-therapeutic response^4^ —and neurobiological endophenotypes, such as brain structure^5^ and postmortem transcriptome profiles^6^. These variations in schizophrenia psychopathology are partially explained by the genetic background of the patients. For example, a family history of schizophrenia is often an indicator of poor treatment outcomes^7^ or socio-functional prognosis, with specific symptoms, including positive, emotional, and disorganized symptoms, being more severe in patients with a family history^8,9^ of psychiatric disorders.

The polygenic risk score (PRS), which assesses heritable predisposition based on multiple common genetic variants with small effects^10,11^, is a valuable tool for quantifying an individual’s risk for schizophrenia by summing the weighted effect sizes of all associated SNPs (single nucleotide polymorphisms) from GWAS (genome-wide association study). PRS has been widely used to evaluate the genomic architecture of schizophrenia compared to other neuropsychiatric diseases and has revealed the genetic characteristics of schizophrenia^11–13^. However, the use of PRS to elucidate the psychopathology of schizophrenia is still challenging. Although PRS is associated with some symptom scales^14,15^ and therapeutic response^16^, it is ineffective in predicting the clinical outcomes of patients^17^ and the onset of schizophrenia^10,18,19^, highlighting that the disorder’s pathophysiology cannot be fully explained by genetic factors alone. This suggests that the heterogeneity of schizophrenia arises not only from various genetic backgrounds but also from other factors, such as postnatal environmental factors. As the pathogenesis of schizophrenia is an alternative to genetic predisposition due to the accumulation of common genetic variants with small effects, rare variants with large effects have been proposed^20^.

Environmental factors, including prenatal and postnatal ones, also play a significant role in the pathogenesis of schizophrenia^21^. For example, maternal infection is a well known prenatal factor increasing risk for schizophrenia^22,23^. As a postnatal factor, some studies have suggested that biological stress such as oxidative stress and inflammation is related to schizophrenia^24,25^. Our previous study revealed that patients with schizophrenia could be grouped based on the expression levels of stress-responsive genes such as superoxide dismutase 2 (*SOD2*), IL-1β (*IL1B*), and TNF-α (*TNF*), with each subgroup displaying distinct characteristics. This suggests that these stress-responsive molecules contribute to the heterogeneity of schizophrenia^26^, with reactive oxygen species (ROS), mainly produced in the mitochondria during energy production, being key factors. ROS include oxygen-based reactive chemicals such as superoxide anion radicals (O_2_^-^), hydroxyl radicals (OH^-^), and hydrogen peroxide (H_2_O_2_). SOD2, an enzyme located within the mitochondrial matrix^27^, plays a crucial role in scavenging superoxide anion radicals. Excessive ROS accumulation, due to mitochondrial dysfunction or excessive stress, increases oxidative stress^28,29^. This accumulation damages cellular components, causing DNA breaks, protein impairment, lipid oxidation^27,30–34^, and shortened telomeres^35^ (a region of DNA sequences at the end of a chromosome). Understanding the impact of oxidative stress may be crucial for understanding the pathophysiology of schizophrenia.

Our previous study, which classified patients with schizophrenia based on stress-responsive gene expression, revealed distinct regulation patterns in DNA repair-related pathways influenced by environmental stress^26^, raising a hypothesis that individuals with reduced genetic vulnerability require strong environmental stress for schizophrenia onset. To validate these findings, the same results must be replicated using genetic factors. This study aimed to stratify patients with schizophrenia by PRS using postmortem brains to examine differential regulation of DNA repair-related pathways between subgroups. Here, we performed genome-wide SNP analysis via microarray on postmortem brain samples from patients with schizophrenia (*n* = 24) and healthy controls (*n* = 48) and calculated PRS using Japanese GWAS to classify 23 patients with schizophrenia and 18 controls into genetically high- and low-risk groups with high and low PRS, respectively. We then compared the gene expression levels measured by RNA-seq in the prefrontal cortex (PFC) between the two groups. Next, we examined the function of the differentially expressed genes (DEGs) using enrichment analysis to identify the differentially regulated pathways between the two groups. Moreover, using *SOD2* expression level as an oxidative stress indicator, as these two depend on the accumulation of oxidative stress^36–38^, we come to raise a hypothesis that the activation of the pathway may be due to oxidative stress. In addition, we investigated whether the expression levels of genes belonging to these pathways were significantly associated with the severity of schizophrenia symptoms before death. The current study is a pioneering study in a small sample size that attempted to stratify the psychopathology of schizophrenia using PRS and revealed gene sets and pathways contributing to the heterogeneity of schizophrenia.

## Patients and methods

### Subjects

Postmortem brain samples from 24 patients with schizophrenia and 48 controls were obtained from the Fukushima Brain Bank of the Department of Neuropsychiatry, School of Medicine, Fukushima Medical University, and the Brain Research Institute, Niigata University. This study was approved by the Ethics Committees of Fukushima Medical University, Niigata University, and Tohoku University Graduate School of Medicine. All procedures were performed after written informed consent was obtained from the next of kin. The demographic information of all samples used for the PRS calculation or subsequent analyses is presented in Supplementary Table 1. The demographic information of the patients and controls included in the analysis of mRNA expression is presented in Table 1. The criteria from the fourth or fifth edition of the Diagnostic and Statistical Manual of Mental Disorders (DSM-IV or 5) were used to diagnose schizophrenia. No patients were diagnosed with substance use disorder. The Diagnostic Instrument for Brain Studies (DIBS) was used to evaluate the antemortem symptoms of each patient with schizophrenia 3 months before their death^39–42^. We classified each item of the antemortem symptoms of DIBS into three subscales: positive symptoms, negative symptoms, and general psychopathology, according to the Positive and Negative Syndrome Scale (PANSS)^43^, Demographic data are presented in Table 1 (samples used for mRNA expression analysis) and Supplementary Table 1 (samples used for PRS calculation).

**Table 1:** Demographic data of patients with schizophrenia and controls.

	Schizophrenia		*p* value (High vs. Low)	Controls	*p* value (High vs. Low vs. CON)
	High-risk schizophrenia patients	Low-risk schizophrenia patients			
**Number of samples**	12	11		21	
**Sex**			1		1^c^
Male	8	7		13	
Female	4	4		8	
**Age at death (years)**^**a**^	67.5 ± 9.3	68.5 ± 13.1	0.84^b^	66.4 ± 17.7	0.93^d^
**PMI (hours)**^**a**^	12.1 ± 12.4	21.2 ± 9.6	0.06^b^	11.5 ± 12.6	0.08^d^
**RIN**^**a**^	6.6 ± 0.6	6.4 ± 0.7	0.40^b^	5.7 ± 0.9	0.002^d^
**DOI (years)**^**a**^	41.1 ± 8.1	36.8 ± 14.6	0.40^b^	-	-
**CP eq (mg/day)**^**a**^	697 ± 761	317 ± 314	0.13^b^	-	-
**PRS**^**a**^	−0.0006 ± 0.0007	−0.0015 ± 0.0003	0.001^b^	-	-

*High vs. low* statistical test conducted between high-risk patients with schizophrenia versus low-risk patients with schizophrenia, *high vs. low vs. CON* statistical test conducted among high-risk patients with schizophrenia versus low-risk patients with schizophrenia versus controls, *PMI* postmortem interval, *RIN* RNA integrity number, *DOI* duration of illness, *CP eq* chlorpromazine equivalent of antipsychotics.

^a^Data are reported as mean ± standard deviation.

^b^Welch’s *t*-tests.

^c^Fisher–Freeman–Halton exact test.

^d^ANOVA.

### Genotyping, imputation, and PRS calculation

Genotyping, imputation, and PRS calculations were performed as previously described^44^. Briefly, we extracted genomic DNA from the frozen cerebellum or occipital cortex and determined genotypes using Infinium Human Exome-12 v1.2, HumanCoreExome-24 v1.0, and BeadChip on an iScan system (Illumina, Tokyo, Japan). Genotyping was performed in 24 patients with schizophrenia and 48 controls. The following criteria were used to select SNPs, leaving 217,405 SNPs: (1) in the autosomal region, (2) with a call rate of >90%, and (3) not duplicated or ambiguous. Genotype imputation was performed on these SNPs using the Michigan Imputation Server (https://imputationserver.sph.umich.edu)^45^, with the 1000 Genomes Project Phase 3 dataset of East Asian ancestry^46^ as a reference panel. SNPs with low imputation quality (*R*^*2*^ < 0.2) were excluded, leaving 10,256,044 SNPs. Quality checks of SNPs and PRS calculations using PLINK v1.9 (http://www.cog-genomics.org/plink/1.9)^47^ and PRSice-2^48^ were performed. For quality checks, SNPs that fulfilled any of the following criteria were excluded: (1) low minor allele frequency (<0.001), and (2) the number of homozygous and heterozygous alleles deviated from the Hardy–Weinberg equilibrium (*p* < 1.0 × 10^–5^). In total, 7,596,758 SNPs were identified. Next, SNPs were pruned based on a pairwise *r*^2^ threshold of 0.25 and a window size of 200 kb, leaving 443,419 SNPs. No samples in this analysis showed high relatedness (>0.125), indicating that there were no related sample pairs among the subjects. Discovery GWAS for calculating PRS was conducted using Japanese samples from publicly available GWAS from the NBDC Human Database of the Japan Science and Technology Agency (hum0197.v3.gwas.v1)^49^. When calculating PRS, we adopted a window size of 250 kb SNPs as clumping thresholds. The significance threshold for SNP inclusion was determined by the *p* value at which the coefficient of determination (R^2^) predicted the onset of schizophrenia as our previous study (*p* = 0.38)^44^. Patients with a PRS at or above and below the median were classified into high and low PRS groups, respectively, and subjected to the following comparisons between the two groups. Controls subjects were also divided into high and low PRS groups based on the control median.

### mRNA expression

RNA extraction and measurements were performed for the 23 patients with schizophrenia and 21 controls (Table 1), as previously described^44^. Total RNA was isolated from the PFC using the AllPrep DNA/RNA Mini Kit (Qiagen, Tokyo, Japan). RNA purity was evaluated based on the RNA integrity number (RIN) using an Agilent 2200 TapeStation (Agilent, Santa Clara, CA, USA). The poly (A) fraction was isolated from total RNA, fragmented, and used to synthesize double-stranded (ds) complementary DNA (cDNA). The ds-cDNA fragments were processed for adaptor ligation, size selection (for 200 bp inserts), and amplification to generate cDNA libraries. These libraries were subjected to paired-end 2 × 101 bp sequencing on a HiSeq 4000 platform using a HiSeq 3000/4000 SBS Kit (Illumina, Tokyo, Japan). We filtered genes with low expression using the filterByExpr function and calculated log2 fold changes in gene expression between patients with schizophrenia with different risks using R and edgeR package^50^, with the following covariates: sex, age, postmortem interval (PMI), and RIN. Owing to the relatively small sample size, continuous covariates were factored into bins, as in previous studies^44,51^. Age, PMI, and RIN were categorized into groups of 10 years, by intervals of 5 h and 1 unit, respectively. To verify the robustness of the DEGs analysis by edgeR, we also compared gene expression levels using quality surrogate variable analysis (qSVA) framework, a valid method for removing the effects of degradation from RNA-seq data, using qsvaR and limma package^52,53^. We used the top 1000 transcripts which were determined to be effective in removing degradation effects in the qsvaR package. The number of surrogate variables retained was calculated as 19 by the function k_qsvs() in the package qsvaR. In this study, we denoted the ratio of gene expression levels using the high-risk group as the reference (low-risk/high-risk). The Benjamini–Hochberg procedure was applied, and FDR < 0.05 was considered as DEGs. We also calculated the mean difference with 95% CI and cohen’s *d* as the effect size. To evaluate the risk for false negatives due to the limited sample size, we estimated statistical power based on mean read counts, dispersion, |log₂FC| ≥ 1.5, and FDR < 0.05 by the package RnaSeqSampleSize and its function est_power(). Additionally, we conducted Gene Set Enrichment Analysis (GSEA)^54^ aiming to determine whether gene expression profiles were related to oxidative-related gene ontology pathways. Genes were ranked according to the degree of differential expression, and subsequently predefined gene sets were tested using a local version of the GSEA analysis tool (http://www.broadinstitute.org/gsea/index.jsp). The Benjamini–Hochberg procedure was applied in GSEA.

### Total telomere and telomere G-tail analyses

Measurement of total telomere length and telomere G-tail length using a hybridization protection assay in the PFC was performed on 21 patients with schizophrenia and five controls, with the same samples used for mRNA expression analyses. The detailed protocol for the hybridization protection assay has been described previously^55,56^. In summary, the phenol–chloroform extraction method was employed to isolate genomic DNA from brain tissue. The G-tail telomere length was measured using 1 μg of non-denatured genomic DNA, whereas total telomere length was assessed using 0.2 μg of denatured genomic DNA. This analysis was conducted using an automated system, the JANUS Automated Workstation, in a 96-plate format, coupled with an EnVision Multilabel Plate Reader (PerkinElmer, Massachusetts, U.S.A.). To ensure accuracy, all samples were evaluated in triplicate and HeLa cell genomic DNA served as a control to correct for inter-assay variability.

### Statistical analysis

Potential confounders in demographic data were compared among patients with schizophrenia (high and low risk) and controls. The Fisher–Freeman–Halton exact test was used for categorical variables (sex), and analysis of variance (ANOVA) was used for the continuous variables (age, PMI, and RIN) of patients with schizophrenia and controls, whereas Welch’s *t*-test was used for the other continuous variants (duration of illness [DOI], chlorpromazine equivalent dose [CP eq], and PRS) between high- and low-risk patients. We also compared PRS between patients with schizophrenia and controls by Welch’s *t*-test. We used multivariable logistic regression models to examine the association between standardized PRS and stress-response subgroups derived from our previous research^26^. The multivariable logistic regression models included age and sex as covariates. We calculated Tjur’s R^2^ to assess model fit, odds ratio (OR) and 95% confidence interval (CI) to assess effect size. Enrichment analyses of the DEGs were performed using the Reactome pathway database by “ReactomePA” package in R^57^ and GO and KEGG pathway database by the Database for Annotation, Visualization, and Integrated Discovery (DAVID). Network visualization was conducted using Cytoscape^58^. We compared the expression level of *SOD2*, telomere length, length of telomere G-tail, and expression level of telomerase associated protein 1 (*TEP1*) between patients with different risks and controls using ANOVA, with sex, age, PMI, and RIN as covariates. Age, PMI, and RIN were categorized into groups of 10 years, intervals of 5 h and units of 1, respectively, as with the edgeR analysis. We also compared the expression levels of seven DNA repair-related genes (*BLM*, *BRIP1*, *GEN1*, *RAD51C*, *RAD51D*, *WRN*, and *XRCC2*) between high-risk patients and controls, low-risk patients and controls, and high-risk and low-risk control subgroups using ANOVA. We conducted a Pearson’s correlation test between the expression levels of DNA repair-related genes and *SOD2*. We conducted Spearman’s rank test and multiple regression analyses to assess associations between the expression levels of DNA repair-related genes, SOD2, general psychopathology scale, positive symptoms scale, and negative symptoms scale. Multiple regression analyses included age, sex, PMI, RIN, case duration, and CP eq as covariates, excluding two patients due to the unavailability of CP eq. To evaluate the risk for false negative due to the limited sample size in correlation analyses, we conducted a power analysis for Spearman’s rank correlation using package pwr and its function pwr.r.test() with our observed correlation coefficient (*r* = 0.45) and an alpha level of 0.05. For all analyses, *p* < 0.05 indicated statistical significance. For multiple comparisons in correlation analyses and multiple regression analyses, we used the Benjamini-Hochberg method. R (version 4.3.1) and edgeR package (version 3.3.6) were used for statistical analysis.

## Results

### Classification of patients with schizophrenia by PRS

**Fig. 1:**
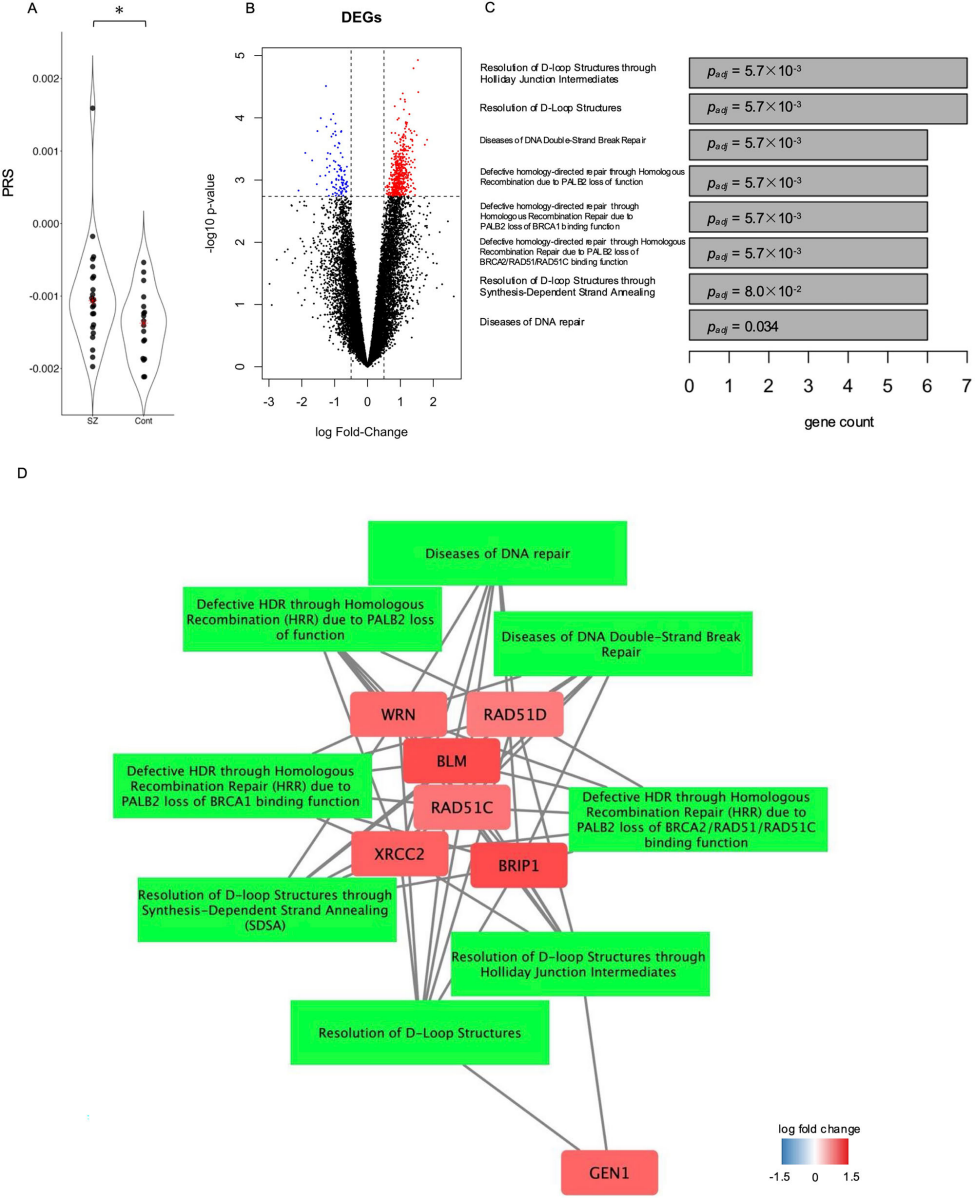
Analyses on DEGs between patients with schizophrenia with different risks. **A** The violin plot shows the distribution of the PRS of patients with schizophrenia and controls. The red mark represents the median. **B** The volcano plot shows changes in gene expression levels between patients with high and low genetic risks. DEGs (FDR < 0.05) are highlighted in red (Fold-Change > 0) and blue (log Fold-Change < 0). **C** Bar plots show the significantly enriched DEG pathways. **D** The network shows the relationships between enriched pathways and DEGs belonging to those pathways. Node color indicates the fold change. SZ schizophrenia, Cont controls, *p* < 0.05*.

Based on the PRS, we divided patients with schizophrenia into two groups: those with PRS higher (*n* = 12) and lower (*n* = 11) than the median (Fig. 1A). PRS was significantly higher in patients with schizophrenia than controls (*p* = 0.04). The demographic data of the two groups and controls are presented in Table 1. Although no significant differences were observed in age at death, sex, PMI, and CP eq among the groups, RIN was significantly different between patients with schizophrenia and controls. We investigated whether PRS is associated with stress-response subgroup investigated in our previous research^26^. Since the 23 patients in the current analyses were all involved in our previous research^26^, we calculated the overlap rate between stress-response subgroup and PRS stratification. Notably, among 12 patients with high PRS, 10 were low stress-responsive group (83%), while five patients were with low PRS among seven patients in high stress-responsive group (71%). In addition, a multivariable logistic regression model adjusting for age and sex revealed a trend-level association between standardized PRS and the stress-response subgroup (Tjur’s *R*^2^ = 0.363, OR = 0.11, 95%CI = 0.01–1.32, *p* = 0.08).

### DEGs between high and low-risk patients with schizophrenia

After comparing the PFC mRNA expression levels between patients with schizophrenia with high and low risk of disease onset, we identified 584 DEGs in edgeR analysis (Supplementary Table 2). Among them, 96 genes showed lower expression levels, whereas 488 genes showed higher expression levels in low-risk patients than in high-risk patients (Fig. 1B). The estimated power to detect differentially expressed genes was 0.69. As a result of enrichment analyses using Reactome, these DEGs were significantly enriched in DNA repair-related pathways, including “Resolution of D-loop Structures through Holliday Junction Intermediates,” “Resolution of D-Loop Structures,” “Diseases of DNA Double-Strand Break Repair,” “Defective HDR (homology-directed repair) through Homologous Recombination (HRR) due to PALB2 loss of function,” “Defective HDR through HRR due to PALB2 loss of BRCA1 binding function,” “Defective HDR through Homologous Recombination Repair (HRR) due to PALB2 loss of BRCA2/RAD51/RAD51C binding function,” “Resolution of D-loop Structures through Synthesis-Dependent Strand Annealing (SDSA),” and “Diseases of DNA repair” (Fig. 1C; Supplementary Table 3). Pathways showing significant enrichment, and the genes belonging to these pathways were visualized as networks (Fig. 1D). Seven DNA repair-related genes were highly expressed in low-risk patients with schizophrenia.

Enrichment analyses using GO and KEGG also revealed that DEGs were significantly enriched in DNA repair- or DNA damage-related pathways such as “DNA repair”, “DNA damage response”, “Rad51B-Rad51C-Rad51D-XRCC2 complex”, and “Homologous recombination” (Supplementary Table 4).

To verify these results, we also conducted DEGs analyses and subsequent enrichment analyses using qSVA. The qSVA framework decreased the effect of RNA degradation (Supplementary Fig. 1) and found 1788 genes with *p* < 0.05, while no DEGs were detected after multiple comparison (Supplementary Table 5). A result of enrichment analysis on these 1788 genes using Reactome did not include DNA repair-related pathways (Supplementary Table 6). On the other hand, results using GO and KEGG included DNA repair- or DNA damage- related pathways such as “single-stranded DNA helicase activity” and “DNA damage response” in GO analysis and “p53 signaling pathway” in KEGG analysis (Supplementary Table 7).

### Expression levels of DNA repair-related genes

**Fig. 2:**
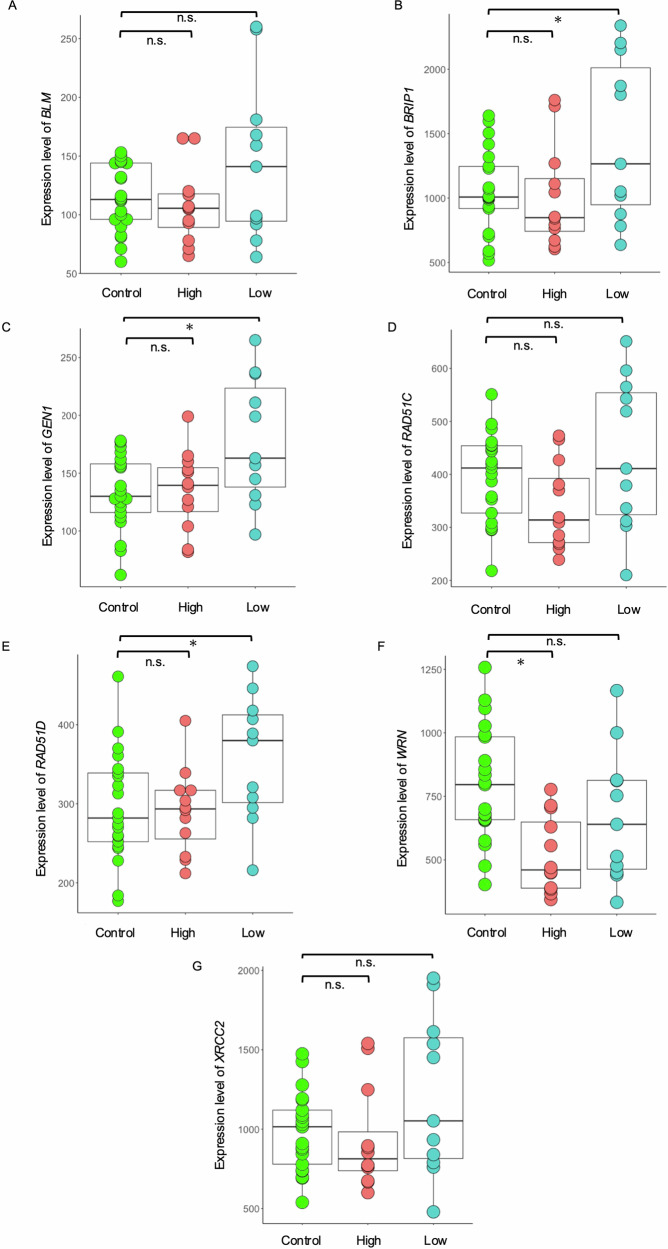
Comparison of DNA repair-related DEGs among schizophrenia patients with different risks and controls. Box plots show the expression levels in patients with schizophrenia and controls of seven DNA repair-related genes differentially expressed between high- and low-risk patients with schizophrenia: **A** *BLM*, **B** *BRIP1*, **C** *GEN1*, **D** *RAD51C*, **E** *RAD51D*, **F** *WRN*, and **G** *XRCC2*. The expression levels were compared between low-risk patients with schizophrenia and controls and high-risk patients and controls, using ANOVA. High, schizophrenia patients with high genetic risk; Low, schizophrenia patients with low genetic risk; n.s., not significant; *p* < 0.05*.

In addition to the comparison between patients with different genetic risks, we compared the expression levels of seven DEGs related to DNA repair (*BLM*, *BRIP1*, *GEN1*, *RAD51C*, *RAD51D*, *WRN*, and *XRCC2*) between high- and low-risk patients with schizophrenia and controls using ANOVA. Since comparison between high-risk patients and low-risk patients was conducted in the DEGs detection, we compared gene expression between controls vs. high- and low-risk patients in this analysis. Four genes (*BLM*, *BRIP1*, *GEN1*, and *RAD51D*) showed significantly higher expression levels in low-risk patients than in controls (Fig. 2A–C, E: *BLM*: *p* = 0.04; *BRIP1*: *p* = 0.008; *GEN1*: *p* = 0.003; *RAD51D*: *p* = 0.01), suggesting that these genes were aberrantly regulated only in patients with a low-risk genetic background. However, except for *WRN*, no significant differences in gene expression were observed between high-risk patients and controls. To determine that upregulation of DNA repair-related pathways is specific to schizophrenia with low PRS, we also conducted PRS stratification and comparable gene expression analyses in control individuals. We divided 18 controls into two groups: those with PRS higher (*n* = 9) and lower (*n* = 9) than the control median (Fig. 1A). We subsequently compared gene expression levels of seven DNA repair-related genes (*BLM, BRIP1*, *GEN1*, *RAD51C*, *RAD51D*, *WRN*, and *XRCC2*) between control subgroups with high and low PRS (Supplementary Fig. 2). No genes showed significant difference between subgroups, supporting that upregulation of DNA repair-related pathways is unique to schizophrenia with low PRS.

### Association between DNA repair-related genes and oxidative stress

**Fig. 3:**
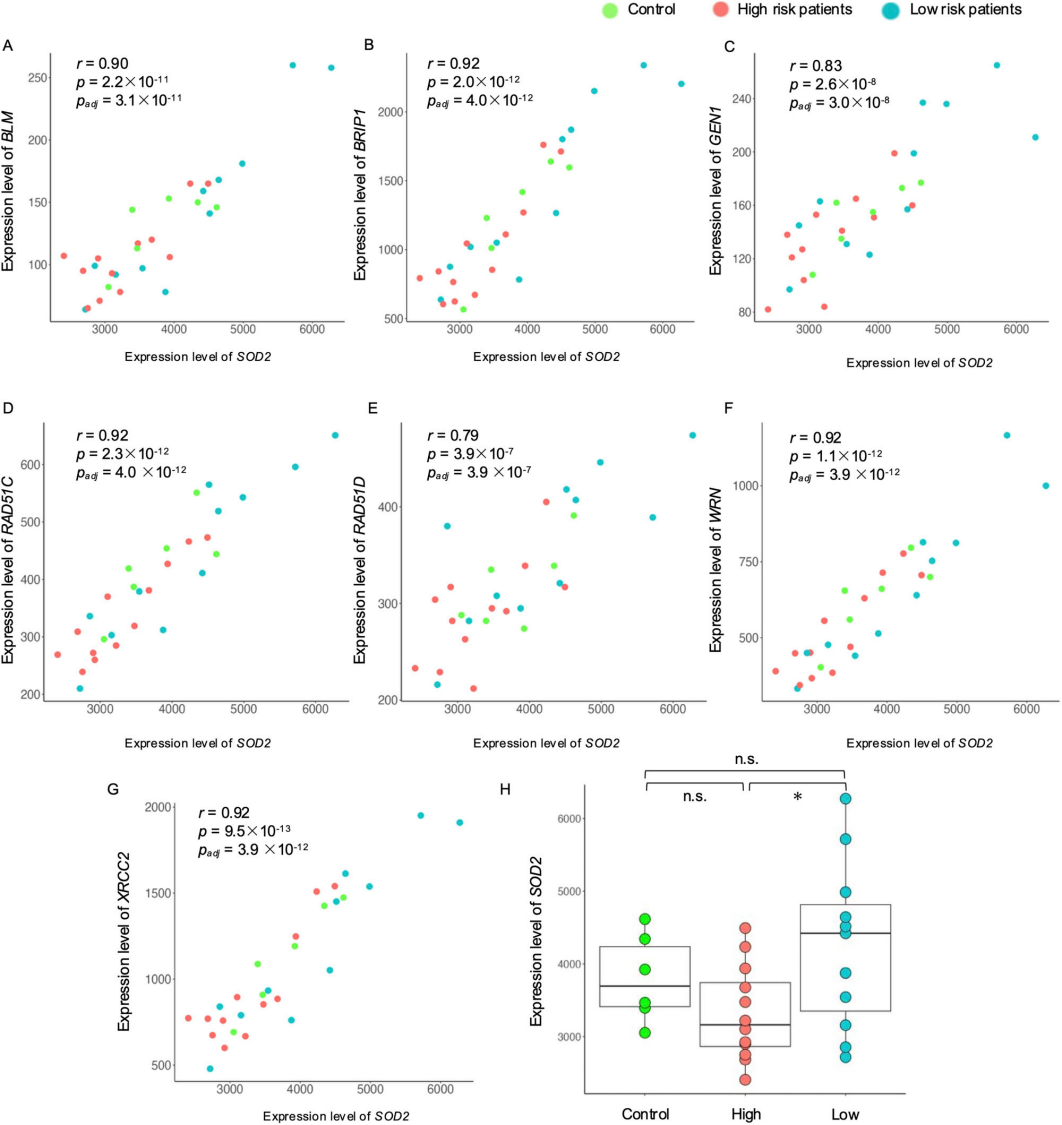
Relationship between DNA repair-related genes and oxidative stress. Scatter plots show significant correlations between expression levels of *SOD2* and **A** *BLM*, **B** *BRIP1*, **C** *GEN1*, **D** *RAD51C*, **E** *RAD51D*, **F** *WRN*, and **G** *XRCC2*. Box plots show significant differences in **H** the expression level of SOD2 between schizophrenia patients with different risks and controls. High, schizophrenia patients with high genetic risk; Low, schizophrenia patients with low genetic risk; n.s., not significant; *p* < 0.05*; p_adj_, adjusted *p* value by the Benjamini-Hochberg method.

To assess the association between DNA repair and oxidative stress, we conducted correlation analyses between the expression of seven DNA repair-related genes (*BLM*, *BRIP1*, *GEN1*, *RAD51C*, *RAD51D*, *WRN*, and *XRCC2*) and oxidative stress (*SOD2* expression) on patients with schizophrenia and controls. There were significant positive correlations between the expression levels of the seven genes and *SOD2* (Fig. 3A–G: *BLM: r* = 0.90, *p* = 2.2 × 10^−11^, adjusted *p* = 3.1 × 10^−11^; *BRIP1*: *r* = 0.92, *p* = 2.0 × 10^−12^, adjusted *p* = 4.0 × 10^−12^; *GEN1*: *r* = 0.83, *p* = 2.6 × 10^−8^, adjusted *p* = 3.0 × 10^−8^; *RAD51C*: *r* = 0.92, *p* = 2.3 × 10^−12^, adjusted *p* = 4.0 × 10^−12^; *RAD51D*: *r* = 0.79, *p* = 3.9 × 10^−7^, adjusted *p* = 3.9 × 10^−7^; *WRN*: *r* = 0.92, *p* = 1.1 × 10^−12^, adjusted *p* = 3.9 × 10^−12^; *XRCC2*: *r* = 0.92, *p* = 9.5 × 10^−13^, adjusted *p* = 3.9 × 10^−12^). Multiple regression analyses adjusting covariates (age, sex, PMI, and RIN) also revealed the significant association between *SOD2* and seven DNA repair-related genes (Supplementary Table 8). *SOD2* expression was significantly higher in low-risk patients than in high-risk patients (Fig. 3H; *SOD2*, *p* = 0.04), while no significant difference was observed between controls and high- and low-risk patients.

To further assess oxidative stress-related molecules in low-risk patients, we used GSEA and tried to evaluate expression trends of other oxidative-stress-related genes. As a result, genes belonging to two pathways which were related to oxidoreductase-related molecular functions (“GOMF_OXIDOREDUCTASE_ACTIVITY_ACTING_ON_THE_CH_NH_GROUP_OF_DONORS_NAD_OR_NADP_AS_ACCEPTOR” [Enrichment score = 0.50, *p* = 0.04, FDR = 0.61], and “GOMF_OXIDOREDUCTASE_ACTIVITY_ACTING_ON_PAIRED_DONORS_WITH_INCORPORATION_OR_REDUCTION_OF_MOLECULAR_OXYGEN_REDUCED_FLAVIN_OR_FLAVOPROTEIN_AS_ONE_DONOR_AND_INCORPORATION_OF_ONE_ATOM_OF_OXYGEN” [Enrichment score = 0.50, *p* = 0.02, FDR = 0.65]; Supplementary Fig. 3) were tended to upregulate in low-risk patients with schizophrenia. Along with SOD2 expression level, those GSEA results may reflect alterations in cellular oxido-redox processes in low-risk patients.

### Correlation between the expression levels of DNA repair-related genes and schizophrenia severity

**Fig. 4:**
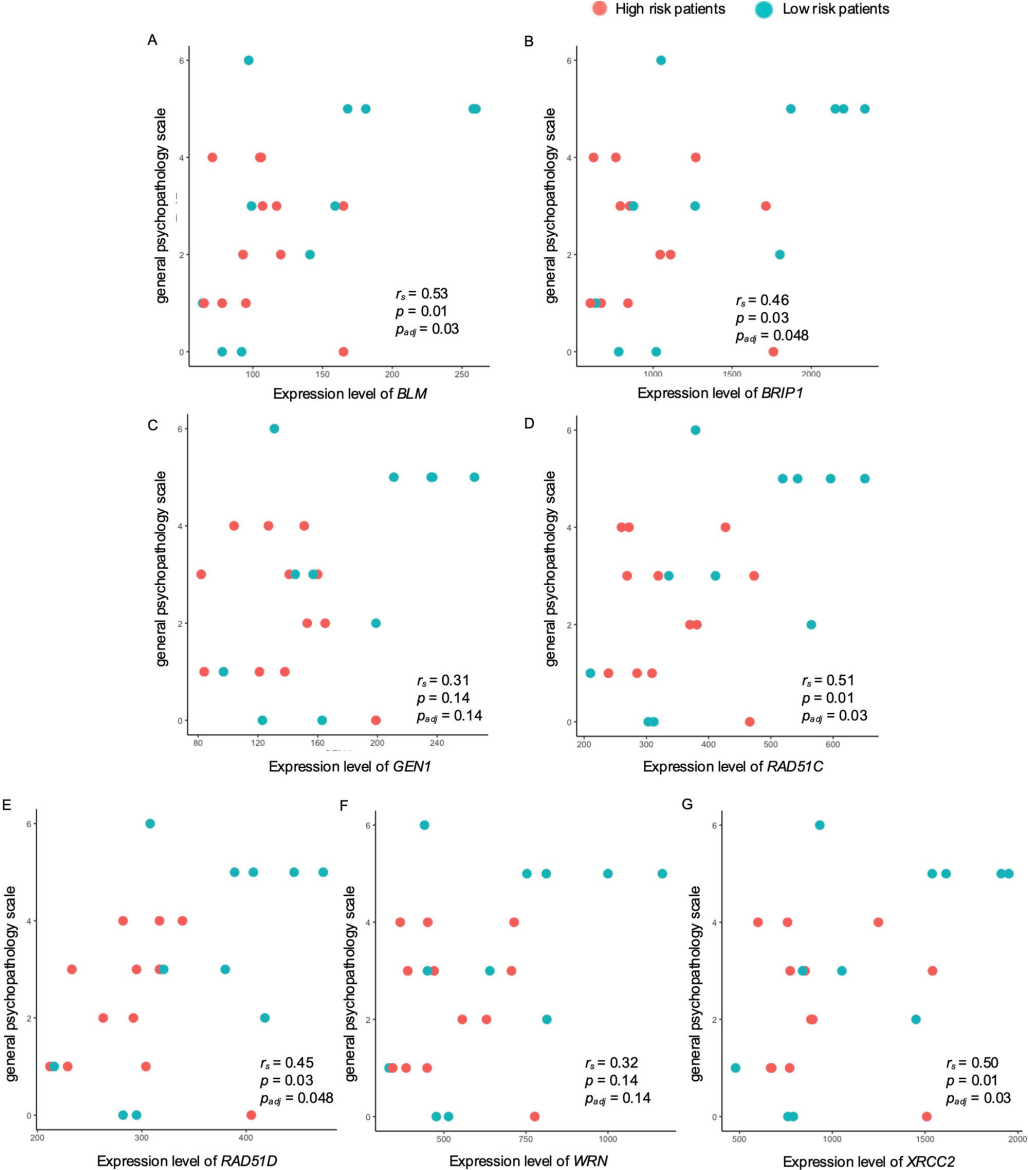
Relationships between DNA repair-related genes and severity of schizophrenia symptoms. Scatter plots show correlations between the general psychopathology scale and expression levels of **A** *BLM*, **B** *BRIP1*, **C** *GEN1*, **D** *RAD51C*, **E** *RAD51D*, **F** *WRN*, and **G** *XRCC2*. p_adj_, adjusted *p* value by the Benjamini-Hochberg method.

Next, we investigated the association between the expression of seven DNA repair-related genes (*BLM*, *BRIP1*, *GEN1*, *RAD51C*, *RAD51D*, *WRN*, and *XRCC2*) and the severity of schizophrenia symptoms using correlation analyses. Among the seven genes, *BLM, BRIP1*, *RAD51C*, *RAD51D*, and *XRCC2* were significantly associated with the general psychopathological scale after multiple comparison (Fig. 4A–E, G: *BLM*: *r*_*s*_ = 0.53, *p* = 0.01, adjusted *p* = 0.03; *BRIP1*: *r*_*s*_ = 0.46, *p* = 0.03, adjusted *p* = 0.048; *RAD51C*: *r*_*s*_ = 0.51, *p* = 0.01, adjusted *p* = 0.03; *RAD51D*: *r*_*s*_ = 0.45, *p* = 0.03, adjusted *p* = 0.048; *XRCC2*: *r*_*s*_ = 0.50, *p* = 0.01, adjusted *p* = 0.03), whereas the other two genes showed no correlation with the general psychopathology scale (Fig. 4C: *GEN1*; *r*_*s*_ = 0.31, *p* = 0.14, adjusted *p* = 0.14; Fig. 4F: *WRN*: *r*_*s*_ = 0.32, *p* = 0.14, adjusted *p* = 0.14).

The positive and negative symptom scales were not associated with gene expression levels (Supplementary Figs. 4 and 5). Similar to the DNA repair-related genes, *SOD2* expression was significantly correlated only with the general psychopathology scale (Supplementary Fig. 6). The estimated power of correlation analyses was 0.60.

To assess effects of covariates on the association between symptoms scale and the seven DNA repair-related genes and *SOD2*, we conducted multiple regression analyses including covariates (age, sex, PMI, RIN, duration of illness, and CP eq) on 21 patients. All eight genes affected general psychopathology scale in the regression model (Supplementary Table 9). After multiple comparison, all seven genes showed significant effects on general psychopathology scale (BLM: multiple *R*^2^ = 0.47, adjusted *R*^2^ = 0.19, adjusted *p* = 0.01; *BRIP1*: multiple *R*^2^ = 0.47, adjusted *R*^2^ = 0.19, adjusted *p* = 0.01; GEN1: multiple *R*^2^ = 0.34, adjusted *R*^2^ = −0.02, adjusted *p* = 0.04; *RAD51C*: multiple *R*^2^ = 0.50, adjusted *R*^2^ = 0.23, adjusted *p* = 0.01, RAD51D: multiple *R*^2^ = 0.31, adjusted *R*^2^ = −0.06, adjusted *p* = 0.048; WRN: multiple *R*^2^ = 0.43, adjusted *R*^2^ = 0.13, adjusted *p* = 0.02; *XRCC2*: multiple *R*^2^ = 0.50, adjusted *R*^2^ = 0.23, adjusted *p* = 0.01). *BRIP1*, *RAD51C*, and *XRCC2* affected positive symptom scale in multiple regression analyses before multiple comparison (*BRIP1*: multiple *R*^2^ = 0.75, adjusted *R*^2^ = 0.62, *p* = 0.04, adjusted *p* = 0.10; *RAD51C*: multiple *R*^2^ = 0.77, adjusted *R*^2^ = 0.65, *p* = 0.02, adjusted *p* = 0.10, *XRCC2*: multiple *R*^2^ = 0.75, adjusted *R*^2^ = 0.62, *p* = 0.02, adjusted *p* = 0.10). No genes affected negative symptom scale in multiple regression analyses. Results of multiple regression analyses are shown in (Supplementary Table 9).

### Comparison of the length of telomere and its G-tail between high- and low-risk patients with schizophrenia

**Fig. 5:**
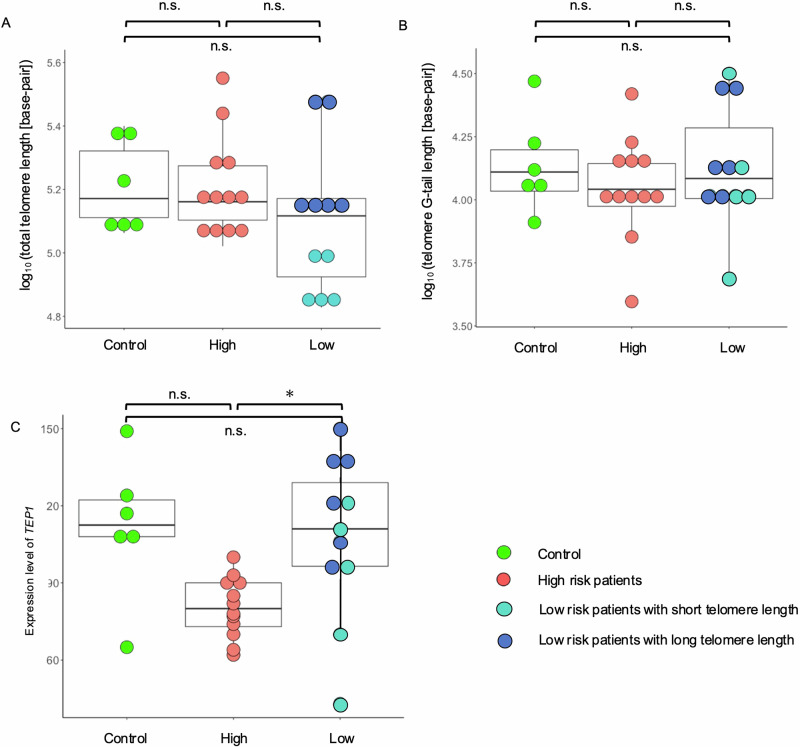
Comparison of telomere and G-tail lengths. Box plots show insignificant differences in **A** telomere length and **B** G-tail length. **C** Significant difference in the expression level of *TEP1* between schizophrenia patients with different risks. Low-risk patients who had longer telomere length than the median are highlighted. High, schizophrenia patients with high genetic risk; Low, schizophrenia patients with low genetic risk; n.s., not significant; *p* < 0.05*.

Lastly, to determine whether telomere length is associated with the upregulation of DNA repair-related genes, we compared telomere length and G-tail length between patients with schizophrenia at different risks. No significant differences were observed between the groups in terms of the total telomere length or telomere G-tail length (Fig. 5A, B). In contrast, the expression level of *TEP1*, a gene encoding telomerase protein component 1 (TEP1) that extends telomere length^59^, was significantly higher in low-risk patients with schizophrenia than in high-risk patients (Fig. 5C, *p* = 0.003), while no significant difference was observed between controls and low- and high-risk patients. In addition, low-risk patients with telomere lengths longer than the median are expected to express high levels of *TEP1*.

## Discussion

We stratified patients with schizophrenia by genetic risk and found that DNA repair-related pathways were uniquely activated in the postmortem PFC of patients with low genetic risk. The expression levels of seven DNA repair-related genes (*BLM*, *BRIP1*, *GEN1*, *RAD51C*, *RAD51D*, *WRN*, and *XRCC2*) were significantly associated with elevated expression levels of *SOD2*, suggesting that oxidative stress may trigger the activation of DNA repair-related molecules. In addition, five of these DNA repair-related genes were significantly linked to the severity of the general psychopathology scale before death, suggesting that DNA damage and oxidative stress affected the clinical phenotypes of schizophrenia. Our current results revealed that the etiology of schizophrenia differs depending on genetic background. The unique activation of DNA repair and oxidative stress in patients with low PRS may imply the possibility that an excessive stress after birth may be essential for the onset of schizophrenia if the individual has a low genetic risk.

The association between brain function and DNA damage has recently attracted increasing attention. DNA breaks and their repair occur in response to non-physiological, special stressors and under physiological conditions that are essential for neural maturation and synaptic plasticity^60–62^. For example, previous studies have reported that the transcriptional regulation of specific genes after neural activity is maintained by DNA break-induced signaling, including double-strand break repair^62,63^. These studies underscore the value of an adequate balance between DNA breaks and repair, implying that aberrant accumulation of DNA breaks and subsequent upregulation of DNA repair may be related to morbid conditions.

Several reports have suggested that abnormal DNA damage and repair are involved in psychiatric disorders, including schizophrenia^64,65^. For example, specific SNPs in DNA repair-related genes, such as *RAD51D* and *XRCC1*, are associated with the risk of schizophrenia^66,67^. This association between psychiatric disorders and DNA repair-related genes may be due to increased oxidative stress caused by mitochondrial dysfunction, as several studies have linked schizophrenia to ROS accumulation^68,69^ and mitochondrial dysfunction^37,70^. Several proteomic approaches have shown that proteins with altered expression in the postmortem brains of patients with schizophrenia are strongly associated with mitochondrial function and oxidative stress responses^37,71^. A postmortem study revealed lower levels of glutathione peroxidase, an antioxidant enzyme, in patients with schizophrenia^72^. This was confirmed by a meta-analysis of magnetic resonance spectroscopy studies^73^, which reported a significant reduction in glutathione levels in the anterior cingulate cortex of patients with schizophrenia. Taken together, it is possible that mitochondrial dysfunction in patients with schizophrenia causes oxidative stress.

Our previous research subgrouping patients with schizophrenia according to the expression levels of stress-related molecules also revealed that genes associated with DNA repair were upregulated in patients with high-stress responses^26^. This study, which stratified patients by genetic risk, also indicated that oxidative stress and DNA breaks may be involved in schizophrenia, further emphasizing the importance of DNA repair in the pathophysiology of schizophrenia. Notably, low PRS was associated with belonging to the high stress-response subgroup, although it did not reach statistical significance. One possible hypothesis is that environmental risk factors highly contribute to the onset of schizophrenia in individuals with low genetic risk, while those with high genetic risk develop schizophrenia even with little exposure to environmental risk factors. It is possible that upregulation of the DNA repair-related pathways is a reflection of exposure to environmental risk factors and subsequent stress response. Collectively, these studies support our findings that the upregulation of DNA repair may be related to the pathophysiology of schizophrenia in individuals with low genetic risk. More detailed investigations are needed to verify our speculative framework.

This study is the first to link schizophrenia symptoms to DNA repair-related genes and to reveal a direct connection between the pathophysiology of schizophrenia and the balance between DNA damage and its repair. Because the expression levels of some DNA repair-related genes were higher in low-risk patients with schizophrenia than in controls, the elevation in DNA repair may be unique to patients with low genetic risk. It is possible that the degree of oxidative stress in patients with schizophrenia may differ depending on the genetic risk and may affect the clinical symptoms. However, the mechanism by which ROS accumulation and DNA breaks elevate general psychopathology scale remains unclear. One possible hypothesis is that oxidative stress may contribute to DNA breakage and subsequent repair. Previous studies reported that oxidative stress is associated with symptom severity^74,75^. Więdlocha et al. reported that blood oxidative status was significantly related to PANSS-total and -positive score. However, to the best of our knowledge, no studies investigating the association between brain oxidative stress-related molecules and clinical information are available, leaving the relationship between the two unknowns. Notably, the current association among oxidative stress, upregulated DNA repair-related pathways, and clinical severity were findings from correlational analyses, leaving the possibility that current results may be derived from other indirect mechanism such as shared upstream factors. Therefore, further studies are required to establish a causal relationship.

Another possible explanation is the crosstalk potential between DNA repair-related molecules and other pivotal neurodevelopmental pathways. For example, RAD51 inhibits the signal of Netrin-1, a crucial protein for developing axons in neurons^76^. Investigating the precise roles of DNA repair-related molecules would help identify essential pathways other than DNA repair.

It is worth noting that only the general psychopathology subscale shows significant correlations with five DNA repair genes in correlation analyses and seven DNA repair genes in multiple regression analyses. This may indicate that general psychopathology subscale is vulnerable to oxidative stress. This may be because the subscale assesses a wide range of symptoms such as cognitive function, anxiety, and depression, reflecting a broader range of brain dysfunction than positive and negative symptoms. This extensiveness may make general psychopathology scale easily affected by oxidative DNA damage. Specifically, the previous study on long-term hospitalized patients with schizophrenia revealed that plasma markers of oxidative stress were associated with cognitive function scores^77^, indicating the possibility that cognitive function may be susceptible to oxidative.

To validate DEGs detection by edgeR, we also conducted qSVA framework. qSVA framework didn’t yield DEGs after multiple comparisons. This inconsistency suggested that RNA degradation and other technical artifacts may affect the current results. Another possibility is that qSVA approach was too robust to use in DEG detection in the small sample size because it is a method to reduce false positives than the conventional method. While results of enrichment analyses on genes with *p* < 0.05 in qSVA framework included DNA-repair related pathways, qSVA approach did not sufficiently replicate edgeR analysis on the points that no DNA repair-related genes were left after the multiple comparisons and enriched pathways were not inconsistent. Those discrepancies underscore the importance of validation in the future studies. In addition to the DNA repair-related pathways, DEGs detected in the qSVA were associated with pathways potentially related to schizophrenia. For example, they were enriched in “nervous system development” in GO analysis. This is consistent with previous studies reporting that developmental deficits are closely associated with schizophrenia^78,79^.

Telomere length, which is shortened by oxidative stress^80^, may be influenced by the pathophysiology of schizophrenia, as patients with psychiatric disorders, such as schizophrenia and major depressive disorder, have shorter telomere lengths^81,82^. Therefore, we hypothesized that low-risk patients with schizophrenia would have shorter telomeres because of increased oxidative stress and compared telomere length and G-tail length among patients at different risks to verify this hypothesis. However, no significant differences were found, and telomeres were not associated with variations in the genetic background. One possible hypothesis is that TEP1 is activated in patients with schizophrenia with low genetic risk and shortened telomere length due to oxidative stress may be recovered by telomerase. To assess this possibility, further biological experiments are required.

This study had several limitations. First, the sample size included in the analyses of mRNA expression and telomere length was relatively small and the detection power is statistically not sufficient. This small sample size is partly owing to the demanding criteria; information on SNPs, transcriptomes, and clinical data was required. Due to the limited sample size, the estimated power in DEG detection was 0.69 and that was in correlation analyses was 0.60, which were relatively low. Stratification by PRS was also performed based on the median, failing to extract subgroups with extremely high or low PRS unlike previous reports^83,84^. In addition, dividing a relatively small number of patients by the median PRS may not adequately capture characteristics of individuals with truly high and low genetic risk at the population level. Notably, most individuals in the current study exhibited negative PRS, as is often observed in PRS analyses^12^. This suggested that individuals have relatively less risk than those in discovery GWAS. Ideally, replication in an independent and larger cohort, particularly including individuals with extremely high and low PRS, would be essential to validate and generalize the current findings. Second, ROS accumulation and DNA breaks in patients with low genetic risk have not been directly evaluated. Further studies are required to elucidate the relationships between oxidative stress, DNA repair, and schizophrenia. Ideally, the causal pathway of DNA repair and symptom severity should be demonstrated. Association between high expression of TEP1 and telomere length in the low-PRS group also remains a speculative hypothesis. Therefore, more detailed causal relationships should be established through biological interventional study including direct telomerase activity assays, complementary experiments in model systems, or longitudinal telomere tracking of patient with schizophrenia in further investigation. Third, not all antemortem lifestyle factors were included as covariates in the current analyses, partly because lifestyle factors were difficult to evaluate. Future studies would be needed to address these limitations and results of the current study should be cautiously interpreted.

This study successfully stratified patients with schizophrenia based on genetic backgrounds and demonstrated the importance of DNA repair and oxidative stress in the etiology and clinical phenotypes of schizophrenia. The unique activation of DNA repair pathways in patients with low genetic risk contributes to the heterogeneity of schizophrenia and is a key clue for fully elucidating its pathophysiology. A more detailed exploration of DNA repair-related pathways in patients with schizophrenia can potentially advance precise and personalized treatments, specifically targeting molecules associated with oxidative stress.

## Supplementary information

Supplementary Figures

Supplementary Tables
