# Supplementary material for: Upregulation of DNA repair-related genes in the prefrontal cortex of patients with schizophrenia with low genetic risk
Source: Schizophrenia (Heidelb). 2026 Apr 8;12(1):49. doi: 10.1038/s41537-026-00748-9 (PMC13234272; doi:10.1038/s41537-026-00748-9)
Supplement: Supplementary file 1 — Supplementary Figures [file 41537_2026_748_MOESM1_ESM.docx]

**Supplementary Information**

**Supplementary Figures**

**Supplementary Figure 1.**


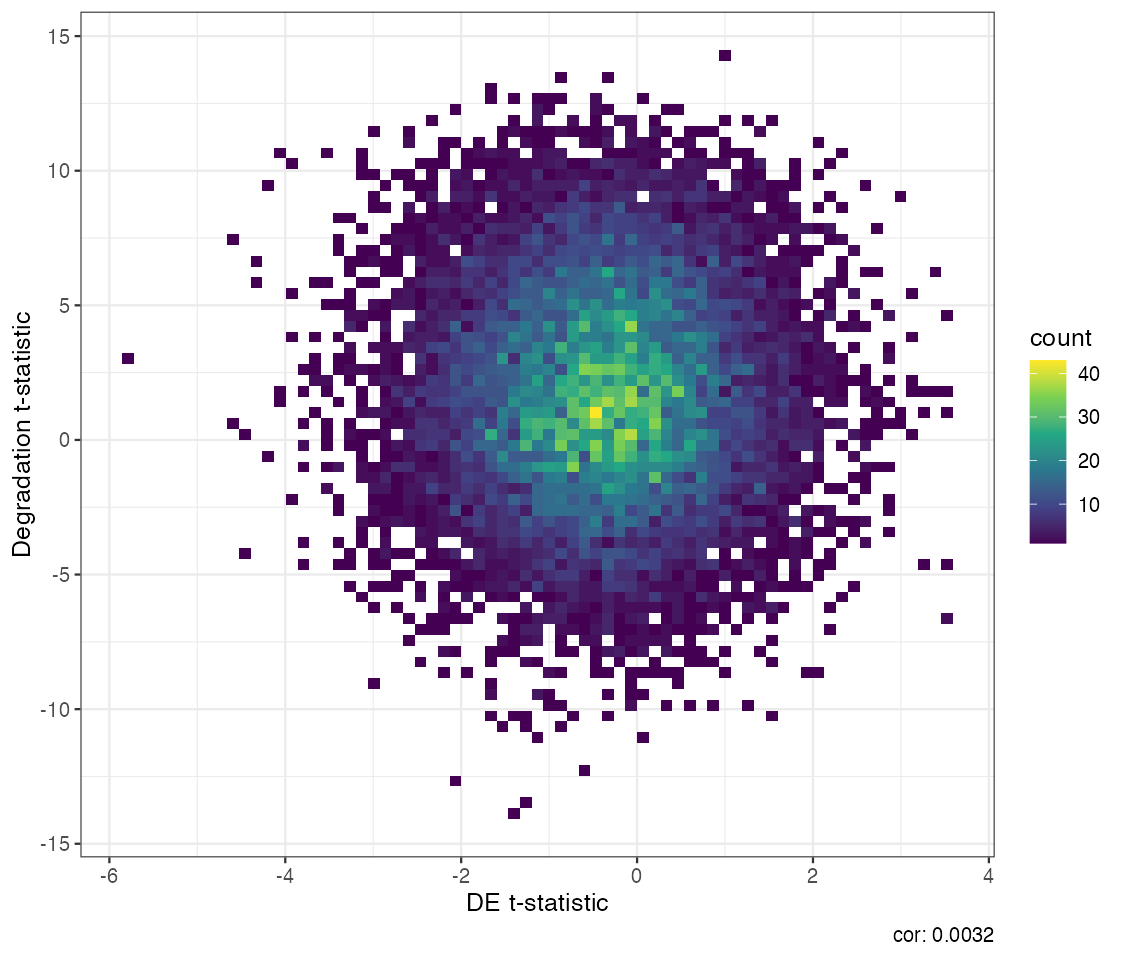


**Supplementary Figure 1:** **Differential expression quality plots for expression differences.** This plot shows that almost no association is observed between the t-statistics from degradation and our differential expression result, indicating that effect of RNA degradation has been well controlled.

**Supplementary Figure 2.**


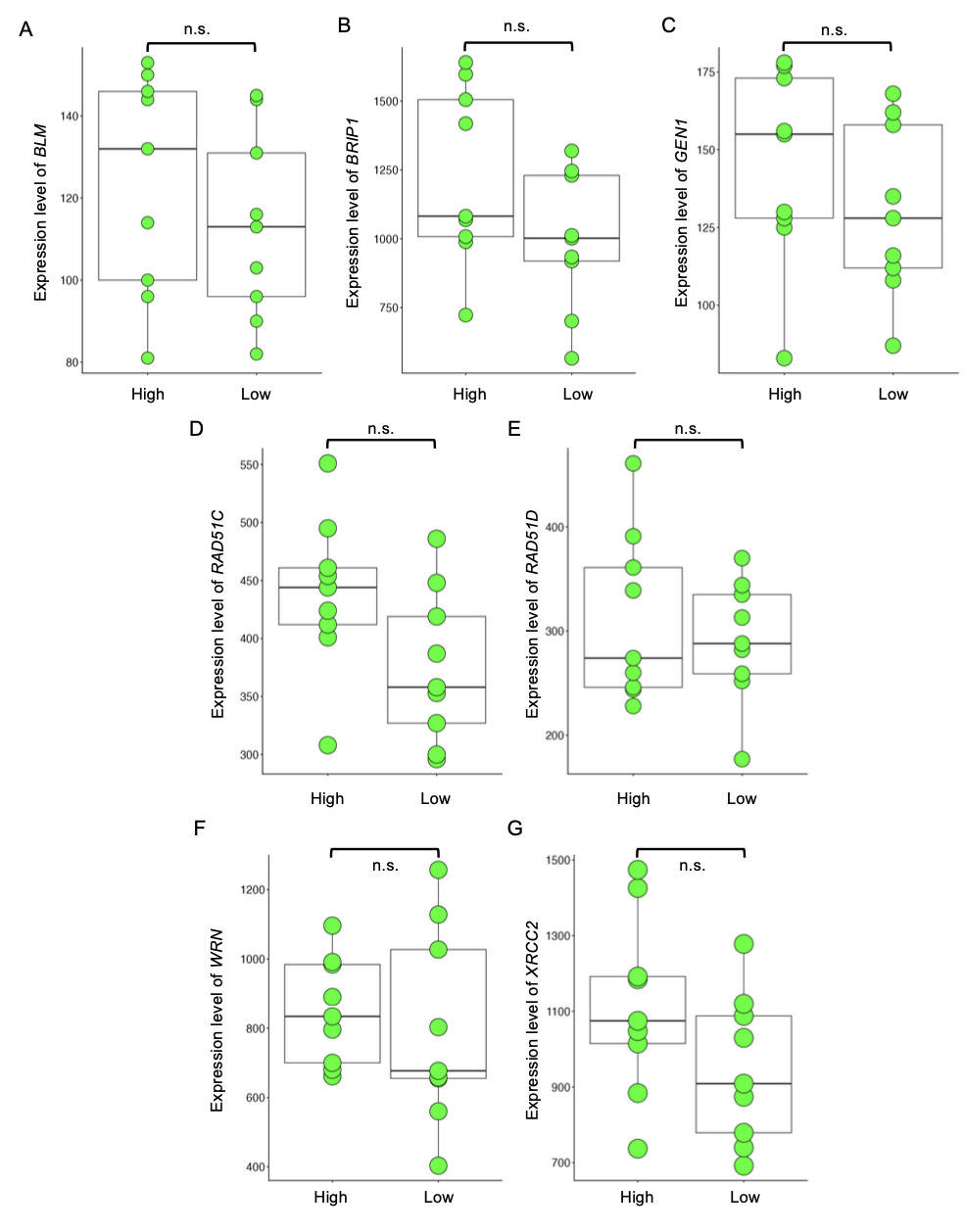


**Supplementary Figure 2:** **Comparison of DNA repair-related DEGs between controls with high and low PRS.** Box plots show the expression levels in patients with schizophrenia and controls of seven DNA repair-related genes differentially expressed between high- and low-risk patients with schizophrenia: **A)** *BLM*, **B)** *BRIP1*, **C)** *GEN1*, **D)** *RAD51C*, **E)** *RAD51D*, **F)** *WRN*, and **G)** *XRCC2*. The expression levels were compared between low-risk and high-risk controls using ANOVA. High, controls with high PRS; Low, controls with low PRS; n.s., not significant.

**Supplementary Figure 3.**

**
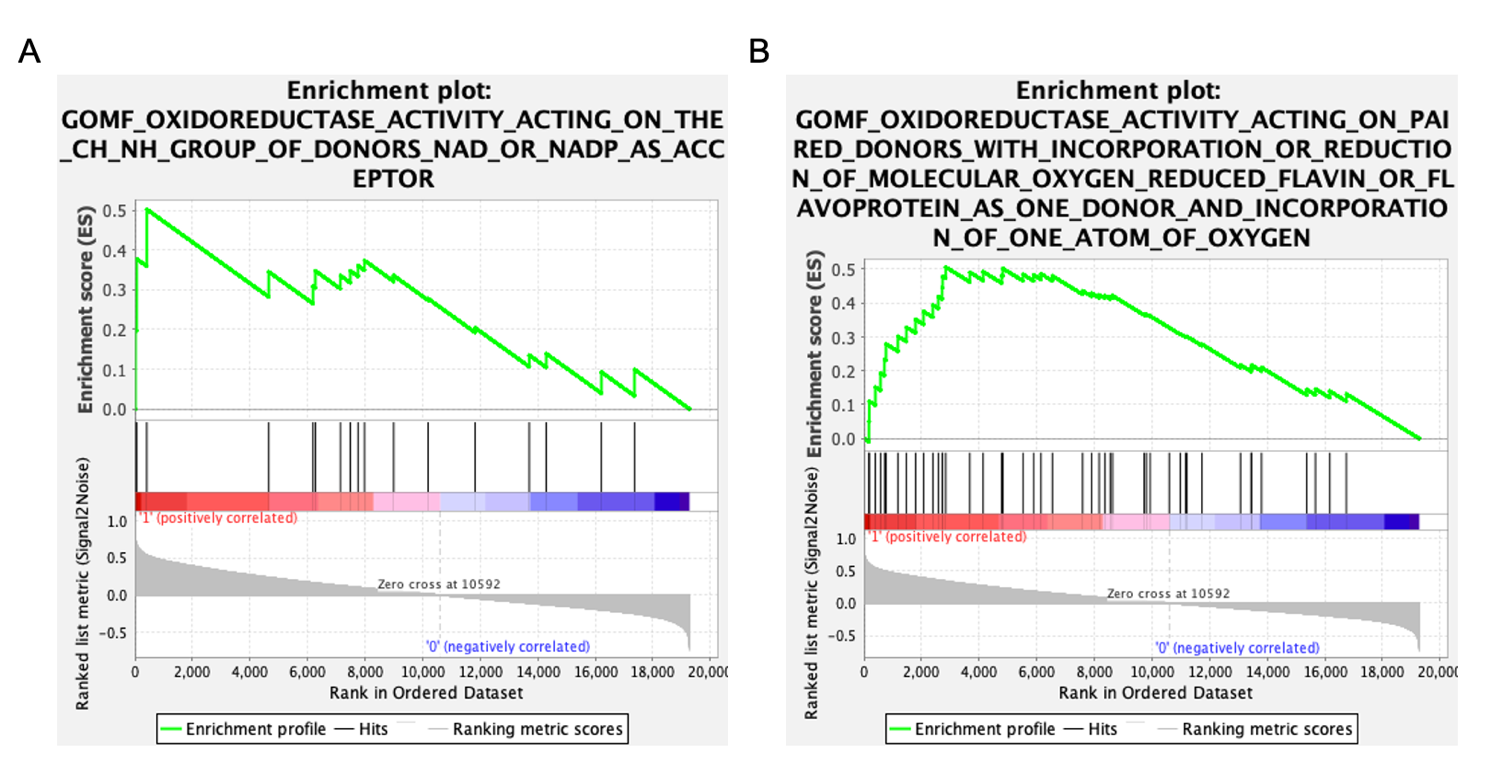
**

**Supplementary Figure 3:** **Results of gene set enrichment analyses of oxido-redox related pathways on DEGs.** The top plot is showing enrichment score for the gene set as the analysis walks down the ranked list, while the bottom plot is showing the ranked list matrix and the location of the genes related to oxido-redox related pathways appear in the DEGs, and the bottom plot showing the value of the ranking metric; **A)** “GOMF_OXIDOREDUCTASE_ACTIVITY_ACTING_ON_THE_CH_NH_GROUP_OF_DONORS_NAD_OR_NADP_AS_ACCEPTOR” and **B)** “GOMF_OXIDOREDUCTASE_ACTIVITY_ACTING_ON_PAIRED_DONORS_WITH_INCORPORATION_OR_REDUCTION_OF_MOLECULAR_OXYGEN_REDUCED_FLAVIN_OR_FLAVOPROTEIN_AS_ONE_DONOR_AND_INCORPORATION_OF_ONE_ATOM_OF_OXYGEN”, B). The plots were generated using a local version of the GSEA analysis tool (<http://www.broadinstitute.org/gsea/index.jsp>).

**Supplementary Figure 4.**

**
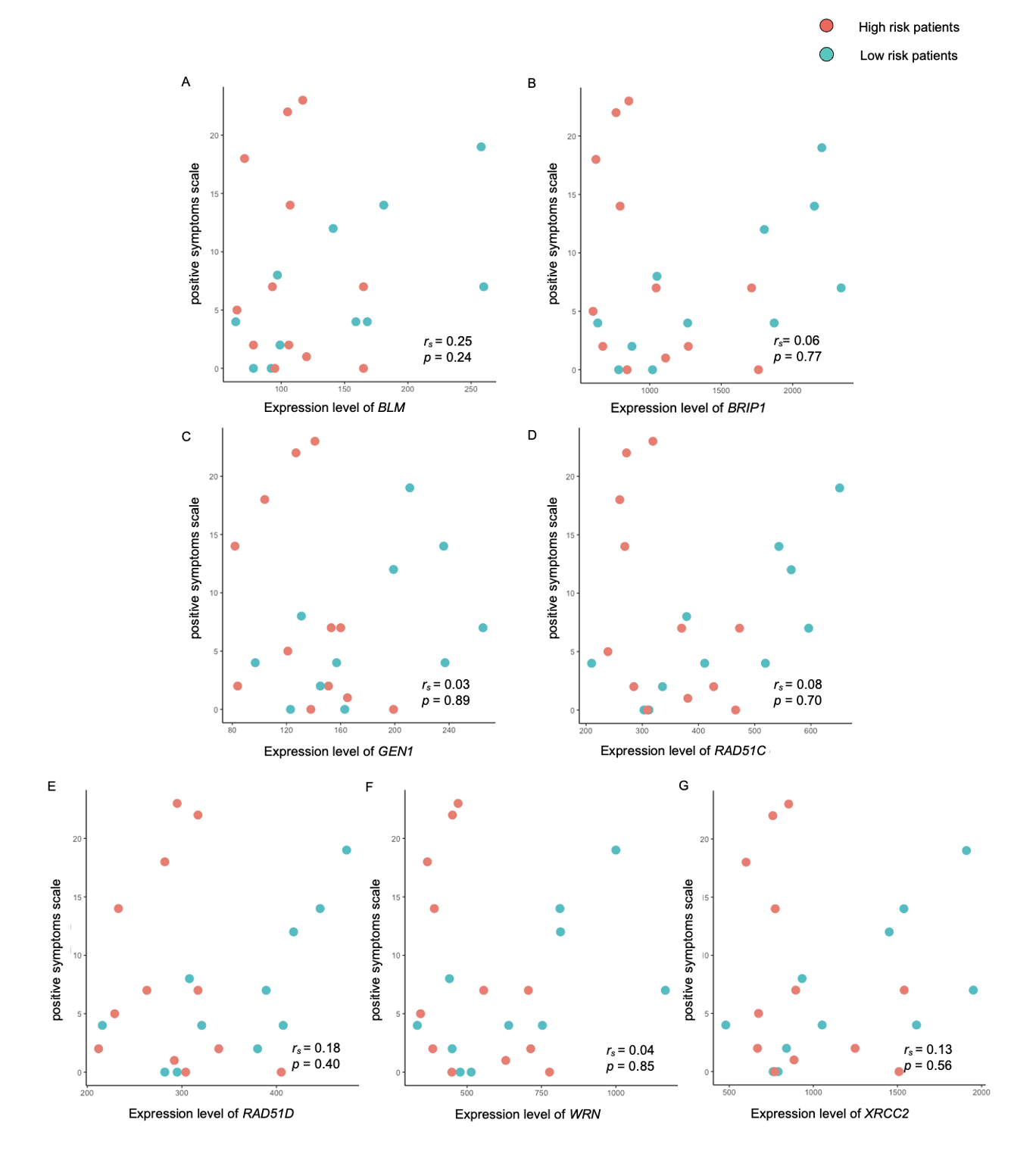
**

**Supplementary Figure 4:** **Relationships between DNA repair-related genes and positive symptoms scale.** Scatter plots show correlations between positive symptoms scale and expression level of **A)** *BLM*, **B)** *BRIP1*, **C)** *GEN1*, **D)** *RAD51C*, **E)** *RAD51D*, **F)** *WRN*, **G)** *XRCC2*.

**Supplementary Figure 5.
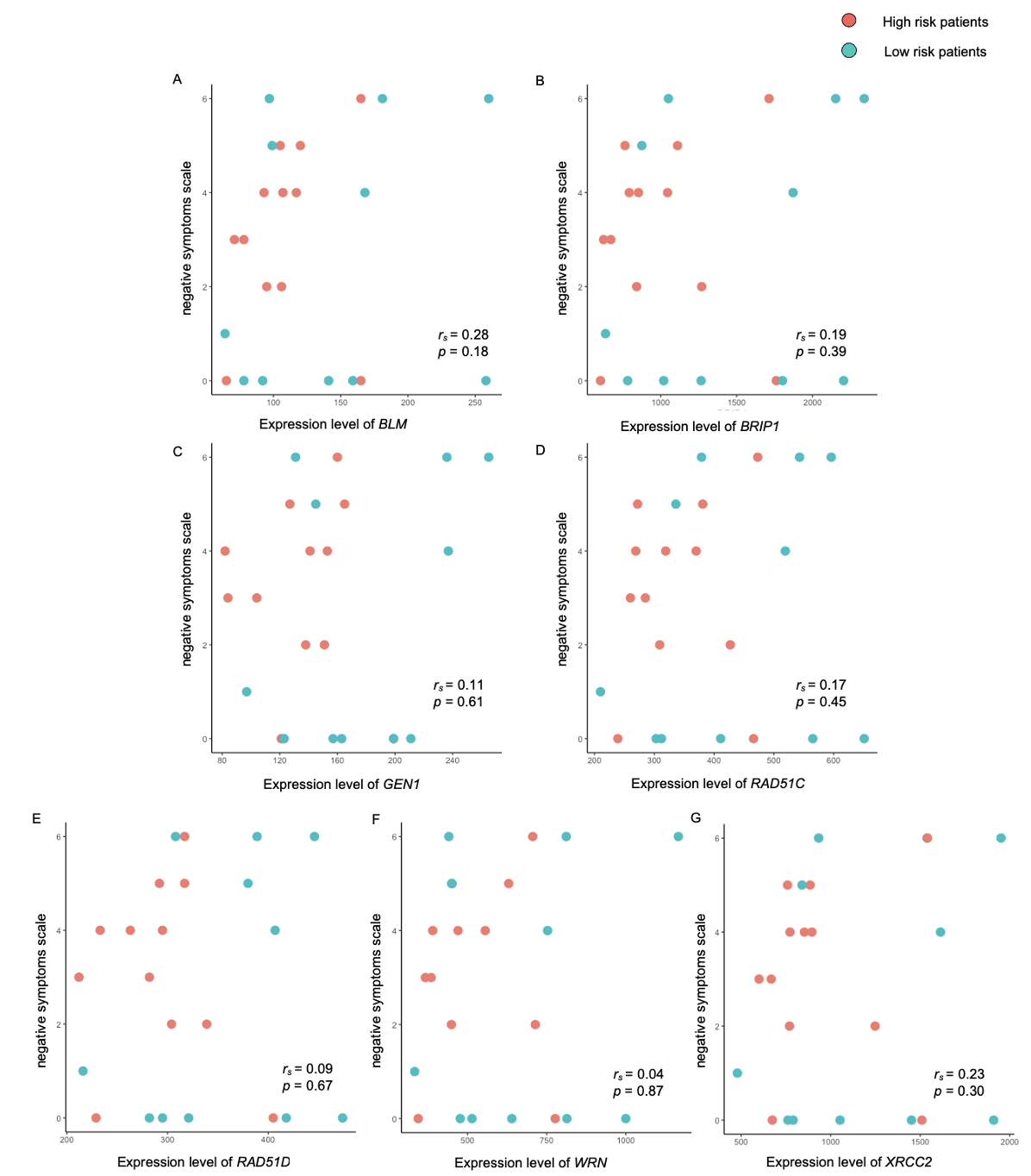
**

**Supplementary Figure 5:** **Relationships between DNA repair-related genes and negative symptoms scale.** Scatter plots show correlations between positive symptoms scale and expression level of **A)** *BLM*, **B)** *BRIP1*, **C)** *GEN1*, **D)** *RAD51C*, **E)** *RAD51D*, **F)** *WRN*, **G)** *XRCC2*.

**Supplementary Figure 6.**


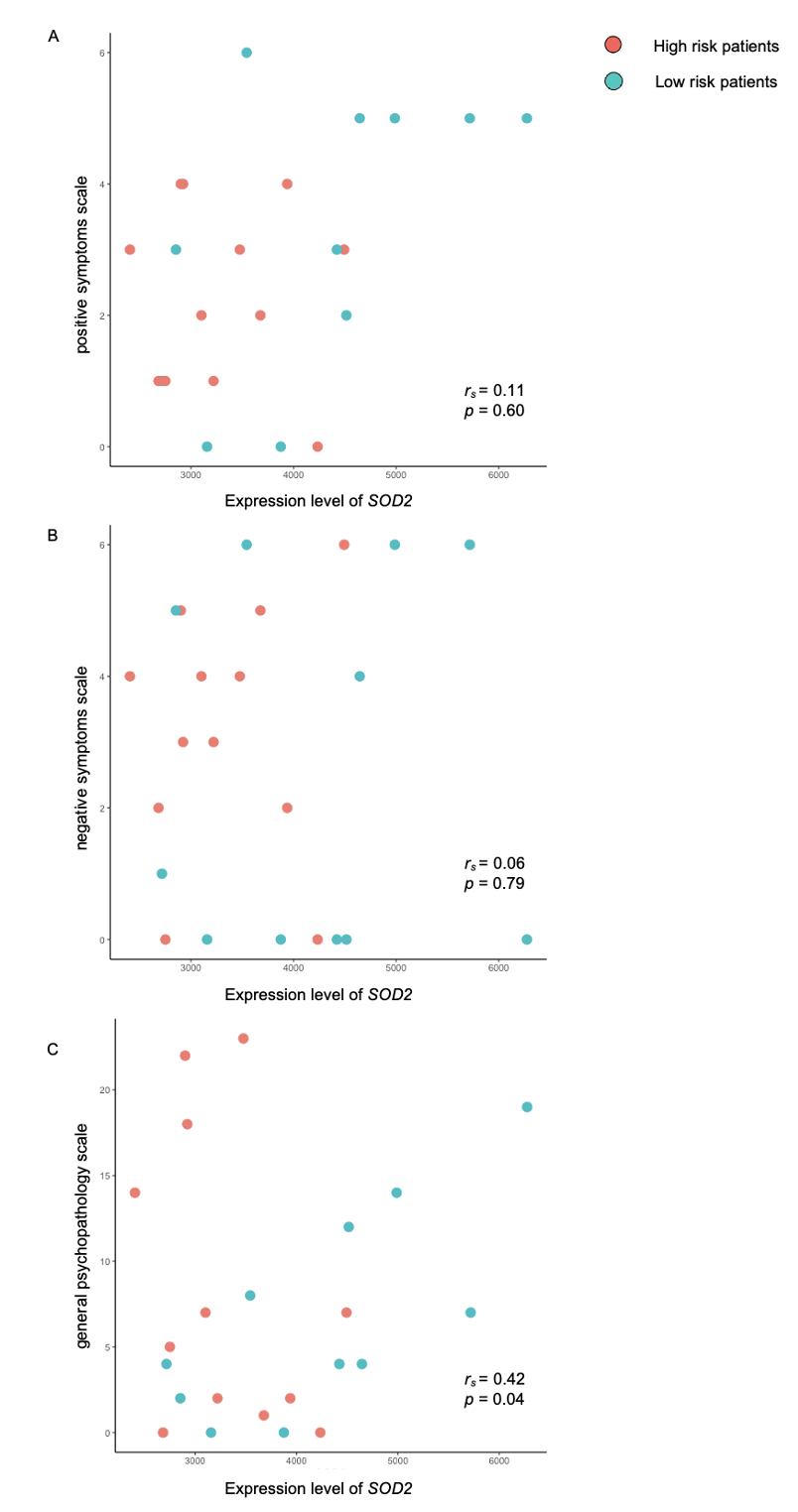


**Supplementary Figure 6: Relationships between expression level of SOD2 and DIBS score.** Scatter plots show correlations between expression level of SOD2 and **A**) positive symptom scale, **B**) negative symptom scale, and **C**) general psychopathology scale.
